# Colistin sulfate versus polymyxin B for the treatment of infections caused by carbapenem-resistant *Acinetobacter baumannii*: a multicenter retrospective cohort study

**DOI:** 10.3389/fphar.2025.1540925

**Published:** 2025-05-14

**Authors:** Hongmei Liu, Li Tang, Liang Zheng, Yuanyuan Fu, Mingjiang Qian, Canghong Ouyang, Na Tao, Shuiping Ou, Yong He

**Affiliations:** ^1^ Department of Pharmacy, The Second Affiliated Hospital of Zunyi Medical University, Zunyi, Guizhou, China; ^2^ College of Pharmacy, Zunyi Medical University, Zunyi, Guizhou, China; ^3^ Department of Intensive Care Medicine, The Second Affiliated Hospital of Zunyi Medical University, Zunyi, Guizhou, China; ^4^ Department of Pharmacy, First People’s Hospital of ZunYi, Zunyi, Guizhou, China; ^5^ Department of Pharmacy, Affiliated Hospital of Zunyi Medical University, Zunyi, Guizhou, China

**Keywords:** carbapenem-resistant *Acinetobacter baumannii* infections, colistin sulfate, efficacy, polymyxin B, safety

## Abstract

**Background:**

Polymyxins are the last line of defense against carbapenem-resistant Gram-negative bacilli infections. However, the efficacy of polymyxins against the independent risk factor of bacterial species is unknown. We aimed to compare the efficacy and safety of colistin sulfate (CS) and polymyxin B (PMB) for carbapenem-resistant *Acinetobacter baumannii* (CRAB) infections.

**Methods:**

We carried out a retrospective multicenter study that included patients with CRAB infections at three tertiary hospitals in Guizhou province, China, from 1 Jan 2020 to 30 Jun 2024. Patients were grouped into the CS group and PMB group. The main outcomes were all-cause 28-day mortality and clinical failure rate. The secondary outcomes included the microbiological cure rate, duration of CS or PMB treatment, and length of hospital stay. Safety was evaluated based on the rates of adverse drug reactions.

**Results:**

A total of 140 patients were included, with 58 patients in the CS group and 82 patients in the PMB group. All-cause 28-day mortality was 32.8% in the CS group and 37.8% in the PMB group (adjusted HR = 0.73, 95% CI 0.38–1.37, *p* = 0.316), and the clinical failure rate was 48.3% and 56.1% (adjusted OR = 0.64, 95% CI 0.29–1.39, *p* = 0.262) in the CS group and PMB group, respectively. There were no significant differences in any of the secondary outcomes. The incidence of acute kidney injury (AKI) in the CS group was lower than that in the PMB group (5.2% vs. 19.5%). Compared to the PMB group, the adjusted odds ratio of AKI was 0.24 (95% Cl 0.06–0.96, *p* = 0.044) for the CS group.

**Conclusion:**

Our results suggest that CS is similarly effective to PMB for CRAB infections but it is associated with fewer safety concerns than PMB. This clinical research provides significant information on the efficacy and safety of CS and PMB for CRAB infections.

## Introduction

The emergence and global spread of carbapenem-resistant *Acinetobacter baumannii* (CRAB) pose a significant threat to global public health due to its high mortality rate and extensive drug resistance (GBD, 2019 Antimicrobial Resistance Collaborators, 2022; Antimicrobial Resistance Collaborators, 2022). CRAB is one of the major pathogenic bacteria responsible for healthcare-associated infections, particularly among patients in intensive care units (ICUs) (Jiang et al., 2022). Given CRAB’s resistance to almost all present antibiotics, clinicians face significant challenges in making treatment decisions (Müller et al., 2023). To address this urgent crisis, the World Health Organization (WHO) has listed CRAB as a top-priority pathogen for the development of new antibiotics (Tacconelli et al., 2018). Unfortunately, newer antibiotics such as cefiderocol and eravacycline show no significant advantages in treating CRAB infections (Shields et al., 2023; Rafailidis et al., 2024). Therefore, polymyxins are used in clinical practice as the last line of defense for treating CRAB infections (World Health Organization, 2017; Tamma et al., 2024).

Polymyxins, as polypeptide antibiotics, have been used in clinical practice since 1950s because of their *in vitro* effectiveness against Gram-negative bacteria (Ahmed et al., 2020). Polymyxins primarily bind to lipopolysaccharides on the bacterial cell membrane. Polymyxins disrupt bacterial cells by altering the integrity and structure of their cell membranes (Nang et al., 2021). Polymyxins, including colistimethate sodium (CMS), polymyxin B (PMB), and colistin sulfate (CS), are currently utilized for CRAB infections in clinical practice. CS and CMS, also referred to as polymyxin E, have the same active ingredient. CMS is an inactive prodrug requiring conversion into its active form within the body to exert antibacterial effects. Both CS and PMB can directly kill pathogens as forms of the prototype drug (Nation et al., 2015; Kengkla et al., 2018).

Some studies have already evaluated the efficacy and safety of PMB in treating CRAB. Qiao et al. (2023) assessed the optimal PMB-based combination therapy for CRAB nosocomial pneumonia. The results show that PMB combined with sulbactam is considered a promising therapy option because of the reduced mortality, resulting from the lower dose of PMB and no enhanced risk of nephrotoxicity. Another retrospective study reports that PMB is considered a relatively effective and safety drug for critically ill patients suffering from infections caused by carbapenem-resistant Gram-negative bacteria (CR-GNB) (Qu et al., 2022).

CS was launched only in China after 2018. CS is usually used to treat CRAB in clinical practice. Wu et al. (2024) evaluated the efficacy and safety of CS for patients with hematological diseases caused by carbapenem-resistant organisms. The findings indicate that CS treatment can provide significant clinical effectiveness and microbial responses. Another retrospective cohort study demonstrates that CS may be effective and safe in treating severe infections caused by CR-GNB (Jin et al., 2022). The previously reported PMB-based and CS-based therapies facilitate the physician in making the decision for infection treatment. However, there still is an urgent challenge for clinicians to choose between CS and PMB to maximize benefit when making antibiotic decisions. In nearly a year, only two studies have evaluated the efficacy and safety of CS versus PMB for the treatment of all CR-GNB infections (Wang et al., 2023; Liu et al., 2024), with no significant differences in efficacy. Since polymyxins are considered to have differential anti-microbial efficacy against different species of CR-GNB infections, meaning that bacterial species are independent risk factors for the anti-microbial efficacy of polymyxins (Lu et al., 2021), it is essential to assess the efficacy of polymyxins for given bacteria, especially in the context of the global crisis of CRAB infections.

In this study, a multicenter retrospective cohort study was conducted to compare the efficacy and safety between CS and PMB for the treatment of given bacterial infections selected as CRAB infections. This study aims to collect real-world data on the efficacy and safety between CS and PMB, thus offering practical evidence for clinical medication decisions about the efficacy and safety between CS and PMB for CRAB infections.

## Methods

### Study design and patients

We conducted a multicenter retrospective cohort study in patients with infections caused by CRAB at three centers of tertiary A-level comprehensive university hospitals in Guizhou province, China, from 1 Jan 2020 to 30 Jun 2024. Patients were selected based on the Rational Drug Use Information System of each participating hospital. Furthermore, we collected the detailed clinical data from the Hospital Information System for these patients and applied the predefined inclusion and exclusion criteria to further select the study population.

Patients were included based on the following criteria: 1) hospitalized adults (≥18 years); 2) received intravenous polymyxins (CS or PMB) more than 72 h for CRAB infections; 3) dosage regimens consistent with pharmaceutical instructions or guidelines; 4) known to be susceptible to colistin (MIC ≤2). Patients were excluded based on the following criteria: 1) pregnant patients; 2) patients aged ≤18 years; 3) patients who did not receive adequate CS or PMB treatment (<72 h); 4) patients infected with CRAB resistant to colistin; 5) patients with missing key data; and 6) patients co-infected with COVID-19.

CRAB infections were defined as follows: 1) isolation of *Acinetobacter baumannii* specimens obtained from infection sites; 2) susceptibility testing showing resistance to meropenem (MIC ≥8 mg/L); 3) presence of signs and symptoms associated with infections; and 4) positive laboratory or imaging test results for infections. Interpretation of all susceptibility results was performed according to the European Committee on Antimicrobial Susceptibility Testing (EUCAST) criteria (European Committee on Antimicrobial Susceptibility Testing, 2024).

This retrospective study was conducted in accordance with Good Clinical Practice and the Declaration of Helsinki, with approval from the Ethics Committee of the Second Affiliated Hospital of Zunyi Medical University, and the requirement for obtaining informed consent from participants was waived. This study was reported according to STROBE recommendations (as detailed in Supplementary Table S1).

### Covariates of interest

Clinical data were collected from medical charts, including demographics of sex and age; severity of disease was assessed using Acute Physiology and Chronic Health Evaluation (APACHE) II score (Jacobs et al., 1988) and Sequential Organ Failure Assessment (SOFA) score (Vincent et al., 1996); the severity of the patients’ comorbidities was calculated using the Charlson Comorbidity Index (CCI) (Charlson et al., 1987), underlying diseases, and infection type. We extracted other covariates including mechanical ventilation, invasive procedures, combination of other regiments defined as combination therapy of concomitant use one or more than antibiotics anti-CRAB therapy for at least 72 h, admission in ICU, creatinine clearance, and microbiology data.

### Outcomes

The primary outcomes were all-cause 28-day mortality and clinical failure rate. Clinical failure was considered a composite endpoint occurring under any of the following conditions: 1) persistence or worsening of patients’ baseline clinical signs or laboratory test abnormalities; 2) deterioration of the condition after an initial improvement; 3) death during treatment with CS or PMB for CRAB infections; 4) requirement for rescue therapy of CRAB infection; 5) re-isolation of CRAB from other sources during polymyxin therapy, along with observable clinical signs of infection.

The secondary outcomes included microbiological cure rate, duration of CS or PMB treatment, and length of hospital stay. The microbiological cure was evaluated for the patients whose repeat specimens were obtained and was defined as the complete eradication of the pathogen from the infection site at the end of treatment.

The outcome of safety was evaluated according to the incidence rate of adverse drug reactions (ADRs), including acute kidney injury (AKI), neurotoxicity, skin pigmentation, and constipation. AKI was determined according to the Kidney Disease: Improving Global Outcomes (KDIGO) criteria (Kellum et al., 2013), which is diagnosed under the following conditions: serum creatinine increases by ≥ 0.3 mg/dL within 48 h or reaches ≥1.5 times the baseline values within 7 days.

### Statistical analysis

Continuous variables were expressed as the mean ± standard deviation (SD) or median (interquartile range, IQR), whereas categorical variables were characterized by their frequency or percentage. The chi-square test or Fisher’s exact test was employed to compare categorical variables across different groups, whereas the Mann–Whitney U test or unpaired Student’s t-test was utilized for analyzing continuous variables. Kaplan–Meier curves utilizing the log-rank tests were used to assess differences in survival between the CS and PMB groups. Inverse probability treatment weighted (IPTW) analysis was used to balance the differences of the baseline covariates to control bias factors. Cox proportional hazards regression models were adopted to calculate the hazard ratio (HR) for mortality with the 95% confidence interval (CI). We calculated the odds ratio (OR) for categorical variables and the median difference (MD) for continuous variables. MD with 95% CI was calculated using the Hodges–Lehmann estimator before IPTW adjustment, and a generalized linear model was used to calculate MD with 95% Cl after IPTW adjustment. Logistic regression was employed to determine the OR with the 95% CI. The balance of baseline covariates between CS and PMB was assessed using the standardized mean difference (SMD), with an SMD <0.1 considered indicative of good balance. All tests were two-tailed, and *p* < 0.05 was considered significant. All statistical analyses were performed using R 4.4.1 and IBM SPSS 26 software applications.

## Results

### Baseline demographic and clinical characteristics

**FIGURE 1:**
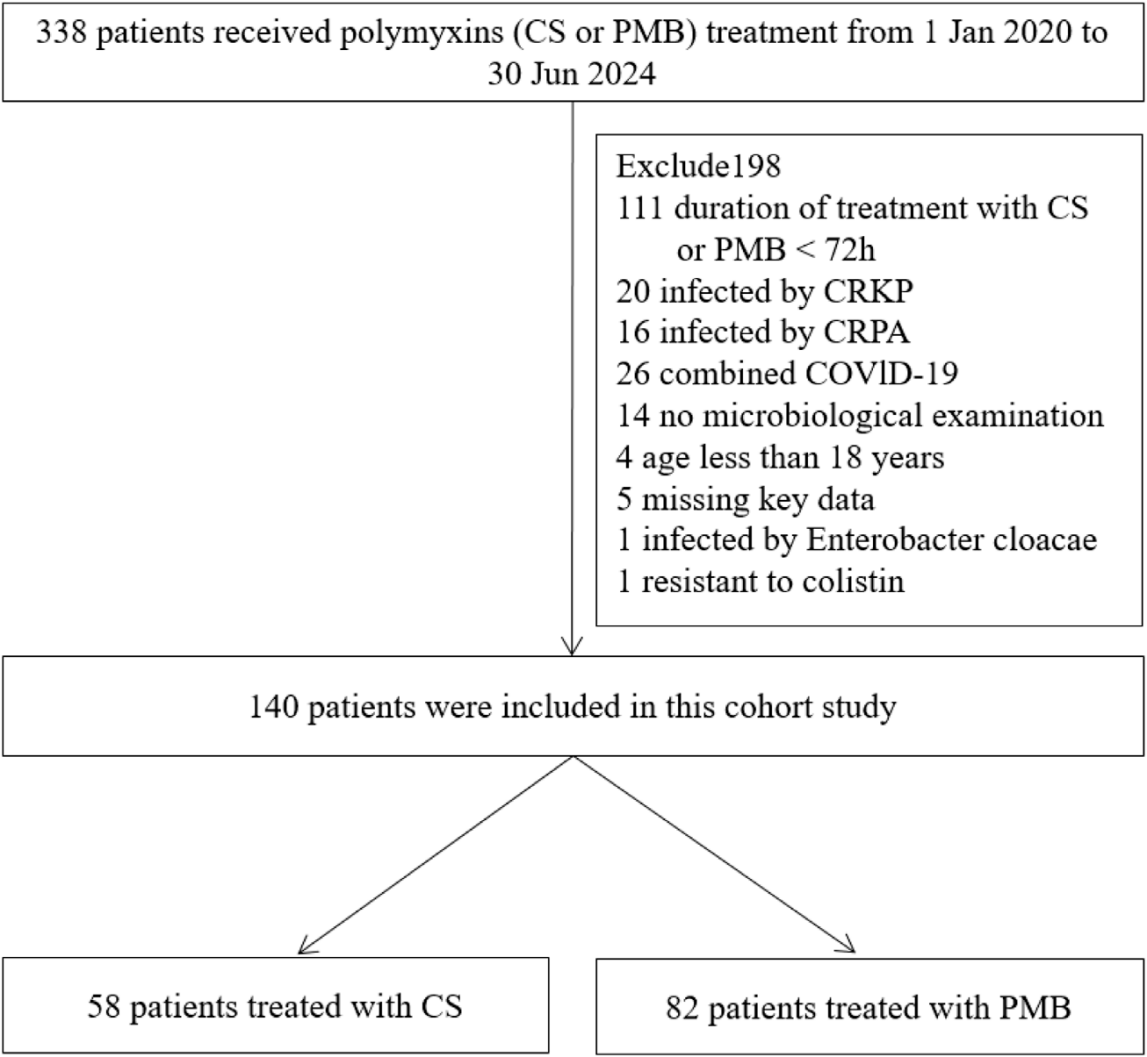
Flow chart of patients. Abbreviations: CS, colistin sulfate; PMB, polymyxin B; CRKP, carbapenem-resistant *Klebsiella pneumoniae*; CRPA, carbapenem-resistant *Pseudomonas aeruginosa*; COVID-19, coronavirus disease.

**FIGURE 2:**
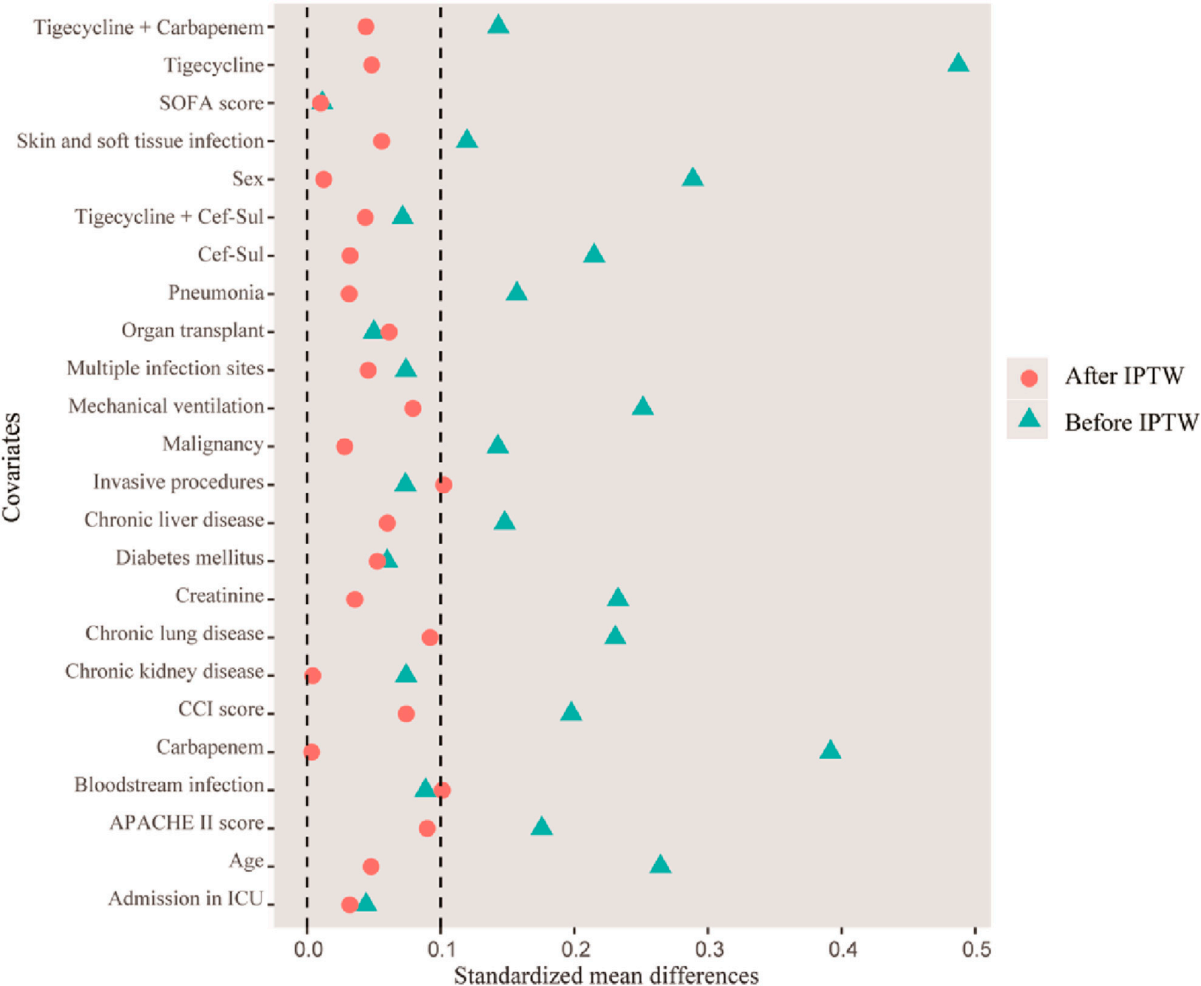
Balance of standardized mean differences before and after IPTW. Abbreviations: IPTW, inverse probability of treatment weighting; SOFA, sequential organ failure assessment; APACHE II, acute physiology and chronic health evaluation II; CCI, Charlson comorbidity index; Cef, cefoperazone; Sul, sulbactam; ICU, intensive care unit.

A total of 338 patients were treated with CS and PMB, of whom 140 were included in the cohort study. A total of 58 patients undergoing CS therapy and 82 patients undergoing PMB treatment were finally included for further evaluation (Figure 1). As illustrated in Table 1, the median age of the patients was 61 years (IQR 45–75), and 98 (70%) patients were male. No significant differences were observed in the APACHE II score, SOFA score, and CCI score in the two groups. Diabetes mellitus, malignancy, chronic lung disease, and chronic kidney disease were the most frequent comorbidities. The most frequent type of infection was 103 (73.6%) patients with pneumonia, followed by 24 (17.1%) patients with multiple infection sites, nine (6.4%) patients with bloodstream infection, and four (2.9%) patients with skin and soft tissue infection. For concomitant antibiotic therapy, 49 (84.5%) patients in the CS group and 64 (75.6%) patients in the PMB group were administered other antibiotics, including cefoperazone–sulbactam, carbapenem, tigecycline, tigecycline plus carbapenem, and tigecycline plus cefoperazone–sulbactam. The proportion of patients in the CS group who received concomitant carbapenem therapy was significantly lower than that in the PMB group (22.4% vs. 40.2%, *p* = 0.041), whereas the proportion of patients who received combined tigecycline in the CS group was higher than that in the PMB group (20.7% vs. 4.9%, *p* = 0.009). After IPTW adjustment, the balance in the baseline characteristics of the variables was improved, and the distribution of standardized mean differences for all variables is shown in Figure 2. Other characteristics of the patients included the length of hospital stay before the diagnosis of CRAB infection, the species and percentage of mixed pathogens, the MIC distribution of the CRAB, the percentage patients receiving nebulized polymyxin, and the number and percentage of patients with septic shock, ARDS, and those who received CRRT. These details are listed in Supplementary Table S1 in the supplementary information. No significant differences were observed in the other characteristics listed in Supplementary Table S1.

**TABLE 1:** Baseline demographics and clinical characteristics of all patients before and after IPTW.

Characteristics	Before IPTW				After IPTW			
	CS (*n* = 58)	PMB (*n* = 82)	SMD	*p* Value	CS (*n* = 56)	PMB (*n* = 80)	SMD	*p*-value
Male, sex	45 (77.6%)	53 (64.6%)	0.289	0.144	38 (67.9%)	55 (68.8%)	0.012	0.953
Age, years, median (IQR)	66 (52–75)	57 (42–72)	0.264	0.126	65 (43–75)	60 (45–74)	0.050	0.718
APACHE II score, mean (SD)	16.6 (6.1)	17.7 (6.6)	0.176	0.311	16.7 (6.7)	17.2 (6.5)	0.090	0.664
SOFA score, median (IQR)	8 (5–12)	8 (5–11)	0.011	0.941	8 (5–12)	8 (6–11)	0.010	0.815
CCI score, median (IQR)	3 (2–5)	3 (1–5)	0.198	0.240	3 (1–5)	3 (1–5)	0.074	0.923
Comorbidity								
Diabetes mellitus	12 (20.7%)	19 (23.2%)	0.060	0.887	13 (23.2%)	17 (21.2%)	0.053	0.794
Chronic lung disease	12 (20.7%)	10 (12.2%)	0.231	0.261	11 (19.6%)	13 (16.3%)	0.092	0.652
Chronic kidney disease	7 (12.1%)	8 (9.8%)	0.074	0.874	5 (8.9%)	7 (8.8%)	0.004	0.980
Malignancy	10 (17.2%)	10 (12.2%)	0.143	0.552	9 (16.1%)	12 (15%)	0.028	0.883
Chronic liver disease	2 (3.4%)	1 (1.2%)	0.148	0.761	1 (1.8%)	1 (1.3%)	0.060	0.676
Organ transplant	1 (1.7%)	2 (2.4%)	0.050	1.000	1 (1.8%)	1 (1.3%)	0.061	0.664
Invasive procedures	55 (94.8%)	79 (96.3%)	0.074	0.990	53 (94.6%)	76 (95.0%)	0.102	0.648
Mechanical ventilation	72 (87.8%)	55 (94.8%)	0.251	0.265	52 (92.9%)	72 (90.0%)	0.079	0.866
Admission in ICU	47 (81%)	65 (79.3%)	0.044	0.966	47 (83.9%)	66 (82.5%)	0.031	0.723
Infection type								
Pneumonia	45 (77.6%)	58 (70.7%)	0.157	0.477	42 (75.0%)	59 (73.8%)	0.032	0.556
Bloodstream infection	3 (5.2%)	6 (7.3%)	0.089	0.873	2 (3.6%)	5 (6.3%)	0.100	0.751
Skin and soft tissue infection	1 (1.7%)	3 (3.7%)	0.120	0.871	1 (1.8%)	2 (2.5%)	0.055	0.694
Multiple infection sites	9 (15.5%)	15 (18.3%)	0.074	0.840	11 (19.6%)	14 (17.5%)	0.046	0.822
Concomitant antibiotic therapy								
Cef–sul	19 (32.8%)	19 (23.2%)	0.215	0.286	16 (28.6%)	21 (26.3%)	0.032	0.868
Carbapenem	13 (22.4%)	33 (40.2%)	0.392	0.042	19 (9.0%)	28 (9.0%)	0.003	0.986
Tigecycline	12 (20.7%)	4 (4.9%)	0.487	0.009	7 (12.5%)	9 (11.3%)	0.048	0.808
Tigecycline + carbapenem	3 (5.2%)	2 (2.4%)	0.143	0.692	2 (3.6%)	2 (2.5%)	0.044	0.793
Tigecycline + cef–sul	2 (3.4%)	4 (4.9%)	0.071	1.000	2 (3.6%)	4 (5.0%)	0.044	0.807
Creatinine, median (IQR)	88 (53–144)	74 (42–106)	0.233	0.087	61 (50–123)	74 (48–105)	0.035	0.865

Abbreviations: CS, colistin sulfate; PMB, polymyxin B; IPTW, inverse probability of treatment weighting; SOFA, sequential organ failure assessment; APACHE II, acute physiology and chronic health evaluation II; CCI, Charlson comorbidity index; Cef, cefoperazone; Sul, sulbactam; ICU, intensive care unit; SMD, standardized mean difference; IQR, interquartile range; SD, standard deviation.

### Primary outcomes

**FIGURE 3:**
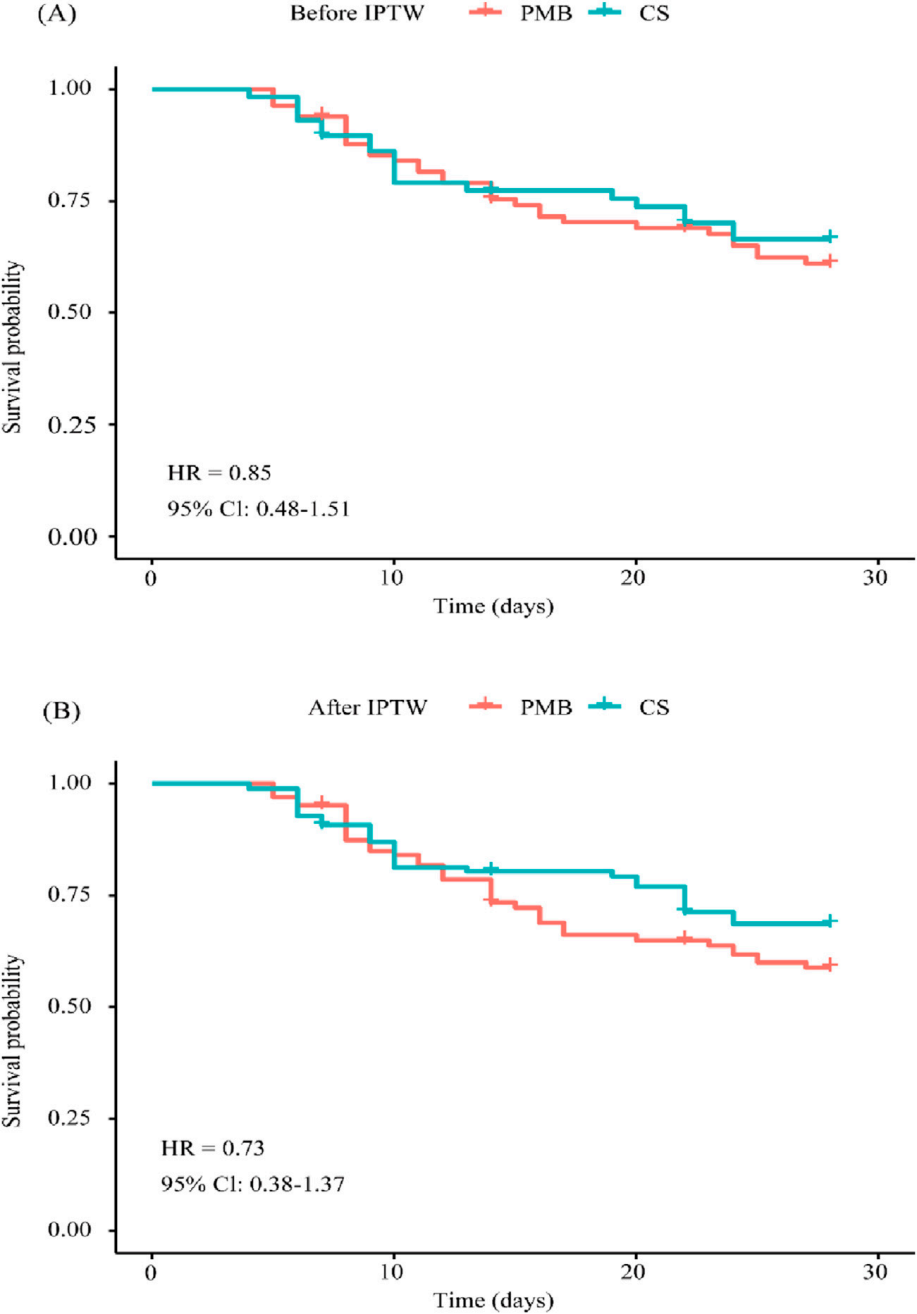
Kaplan–Meier survival curves for patients treated with PMB and CS. **(A)** Before IPTW-adjusted analysis and **(B)** after IPTW-adjusted analysis. Abbreviations: CS, colistin sulfate; PMB, polymyxin B; IPTW, inverse probability of treatment weighting; HR, hazard ratio; CI, confidence interval.

No statistically significant differences were observed in the two primary outcomes between the CS and PMB groups. All-cause 28-day mortality was similar between the CS (32.8%, 19/58) and PMB (37.8%, 31/82) groups (unadjusted HR = 0.85, 95% CI 0.48–1.51, *p* = 0.539), indicating no statistically significant difference. Even after IPTW adjustment, there was no difference in all-cause 28-day mortality between the CS and PMB groups (HR = 0.73, 95% Cl 0.38–1.37, *p* = 0.316). Kaplan–Meier survival curves showed no significant difference in time to death in the two groups both before and after IPTW-adjusted analysis (Figure 3). As listed in Table 2, there was no significant difference in the clinical failure rate between the CS and PMB groups (unadjusted 48.3% vs. 56.1%, OR = 0.73, 95% CI 0.37–1.43, *p* = 0.362). Even after IPTW adjustment, there was no significant difference between the two groups (OR = 0.64, 95% Cl 0.29–1.39, *p* = 0.262).

**TABLE 2:** Comparison of therapeutic efficacy between CS and PMB before and after IPTW.

Outcomes	Before IPTW				After IPTW	
	CS	PMB	*p*-value	HR/OR/MD^a^	*p*-value	HR/OR/MD^a^
	(*n* = 58)	(*n* = 82)		(95% Cl)		(95% Cl)
Primary outcome						
Mortality at 28 days	19 (32.8%)	31 (37.8%)	0.539	0.85 (0.48–1.51)	0.316	0.73 (0.38–1.37)
Clinical failure	28 (48.3%)	46 (56.1%)	0.362	0.73 (0.37–1.43)	0.262	0.64 (0.29–1.39)
Secondary outcome						
Microbiological cure^b^	27 (62.8%)	44 (66.7%)	0.716	0.85 (0.38–1.95)	0.924	1.03 (0.40–2.77)
Duration of CS or PMB treatment, days, median (IQR)	10 (7–15)	9 (6–12)	0.347	1.00 (−1.00–3.00)	0.133	2.00 (−1.00–4.00)
Length of hospital stay, days, median (IQR)	36 (24–57)	44 (27–63)	0.138	−6.00 (−14.00–2.00)	0.511	−5.00 (−14.00–13.00)

^a^ Referenced to the PMB group.

^b^ Only evaluated for the patients with repeating specimens.

Abbreviations: CS, colistin sulfate; PMB, polymyxin B; IPTW, inverse probability of treatment weighting; OR, odds ratio; MD, median difference; HR, hazard ratio; IQR, interquartile range; CI, confidence interval.

### Secondary outcomes

There were no significant differences in the length of hospital stay, duration of CS or PMB treatment, and microbiological cure rate in the two groups. As detailed in Table 2, the microbiological cure rates were 62.8% and 66.7% for the CS and PMB groups, respectively. Compared to the PMB group, the adjusted OR was 1.03 (95% Cl 0.40–2.77; *p* = 0.924) for the CS group. The median lengths of stay in the hospital were 36 days (IQR 24–57) for the CS group and 44 days (IQR 27–63) for the PMB. Compared to the PMB group, the adjusted median difference (MD) was −5.00 (95% Cl −14.00–13.00, *p* = 0.511) for the CS group. The median duration of PMB treatment was 9 days (IQR 6–12) for the PMB group, and the median duration of CS treatment was 10 days (IQR 7–15) days for the CS group. Compared to the PMB group, the adjusted MD was 2.00 (95% Cl −1.00–4.00, *p* = 0.133) for the CS group.

### Safety

The ADR associated with PMB and CS therapy primarily included AKI, followed by skin pigmentation, neurotoxicity, and diarrhea (Table 3). Six (10.3%) patients were observed in the CS group compared with 20 patients (24.4%) in the PMB group (unadjusted OR = 0.36, 95% Cl 0.12–0.91, *p* = 0.041). There still existed a difference after IPTW adjustment (OR = 0.31, 95% Cl 0.12–0.97, *p* = 0.044). Furthermore, we found that the difference in the incidence of ADR between the two groups was attributed to variations in AKI. The incidence of AKI in the CS group was 5.2%, whereas its incidence in the PMB group was significantly higher (19.5%). Compared to the PMB group, the adjusted OR was 0.24 (95% Cl 0.06–0.96, *p* = 0.044) for the CS group. Detailed information on the severity stage of AKI, duration of AKI, and the number of patients that required renal replacement therapy are listed in Supplementary Table S1 (see supplementary information). For the incidence of AKI (Supplementary Table S1), the adjusted absolute risk reduction rate was 0.13 (95% CI 0.03–0.24), and the number needed to harm was estimated to approximately be 8 (95% CI 4–34). The result showed that the CS group had a significantly lower risk of AKI than the PMB group. There were no significant differences in neurotoxicity, skin pigmentation, and diarrhea in the two groups.

**TABLE 3:** Comparison of safety evaluation between CS and PMB groups before and after IPTW.

Safety	Before IPTW				After IPTW	
	CS (*n* = 58)	PMB (*n* = 82)	*p*-value	OR^a^ (95% Cl)	*p* Value	OR^a^ (95% Cl)
Overall	6 (10.3%)	20 (24.4%)	0.041	0.36 (0.12–0.91)	0.044	0.31 (0.12–0.97)
Nephrotoxicity	3 (5.2%)	16 (19.5%)	0.023	0.22 (0.05–0.72)	0.044	0.24 (0.06–0.96)
Skin pigmentation	1 (1.7%)	4 (4.9%)	0.340	0.34 (0.02–2.39)	0.411	0.38 (0.04–3.75)
Neurotoxicity	2 (3.4%)	1 (1.2%)	0.390	2.89 (0.27–63.13)	0.541	1.42 (0.02–7.06)
Diarrhea	1 (1.7%)	0 (0)	NA	NA	NA	NA

^a^ Referenced to the PMB group. NA, not available.

Abbreviations: CS, colistin sulfate; PMB, polymyxin B; IPTW, inverse probability of treatment weighting; OR, odds ratio; CI, confidence interval.

## Discussion

CRAB, as one of the primary pathogens responsible for hospital-acquired infections, is associated with high mortality rates. Due to its broader spectrum of drug resistance compared to other CR-GNB, the available treatment options are extremely limited. As the last line of defense against CR-GNB infections, the efficacy and safety of polymyxins are quite essential for public health. It is urgent to conduct head-to-head research on the efficacy and safety of colistin and PMB (Nation et al., 2015). Furthermore, since bacterial species are considered an independent risk factor for polymyxin efficacy, it is essential to conduct research on the efficacy and safety of PMB and CS for the given bacteria.

In this retrospective cohort study, we select the given bacteria as CRAB since CRAB is one of the major pathogens listed by WHO. We conducted a head-to-head study for CRAB infections between CS and PMB. We find that there is no significant difference in all-cause 28-day mortality, clinical failure rate, microbiological cure rate, duration of CS or PMB treatment, and length of hospital stay in the two groups. CS therapy is similarly effective as PMB therapy for CRAB infections. For the evaluation of efficacy between CS and PMB, only a few reported studies have been reported across all species of CR-GNB. Wang et al. (2023) conducted a retrospective study to compare the efficacy of CS and PMB for all the CR-GNB, including *Klebsiella pneumoniae*, *Pseudomonas aeruginosa*, *Acinetobacter baumannii*, *Escherichia coli*, *Enterobacter cloacae*, and *Acinetobacter junii*. This study also found no significant differences in 28-day mortality (33.3% for CS vs. 39.7% for PMB) and clinical success rate (41.7% for CS vs. 33.8% for PMB). Liu et al. (2024) also conducted a real-world study to assess the effects of CS and PMB for pneumonia treatment caused by all the CR-GNB including *Klebsiella pneumoniae*, *Pseudomonas aeruginosa*, and *Acinetobacter baumannii*. There were no significant differences in good clinical response, 28-day mortality, and all-cause mortality. The previous studies focusing on all CR-GNB did not specifically subgroup bacteria, resulting in a lack of comparative efficacy data only for the given pathogen. In our study, especially for the pathogen of CRAB, the results also show no significant differences in 28-day mortality or clinical failure rates between CS and PMB in treating CRAB infections. This finding further supports and complements the results of existing studies. However, for other important pathogens such as *Klebsiella pneumoniae* or *Pseudomonas aeruginosa*, there are still few studies between CS and PMB. Both CS and PMB have similar mechanisms of action and relatively close pharmacokinetic properties, leading to their similar clinical efficacy (Xie et al., 2022; Yu et al., 2022).

Lu et al. (2021) analyzed the factors influencing the microbiological efficacy of PMB on CR-GNB infections. PMB was found to exert differential microbiological efficacy on the different species of CR-GNB infections. The independent risk factors for PMB microbiological efficacy are considered bacterial species and multiple CR-GNB infections. Another retrospective cohort study was conducted to evaluate the 30-day mortality of PMB treatment using Cox regression analysis. In contrast, to treat *Pseudomonas aeruginosa*, PMB combination with other antibiotics showed a protective effect in treating *Acinetobacter baumannii*, indicating that the efficacy of polymyxins may differ against various pathogens (Rigatto et al., 2015). The microbiological efficacy of CS in treating CRAB currently remains unknown. In our study, the microbiological cure rate in the CS group was 62.8%, which was similar to that of the PMB group (66.7%). Furthermore, the similar results for the length of hospital stay and duration of CS or PMB treatment in both patient groups support the notion that CS and PMB exhibit similar efficacy in treating CRAB infections.

The safety of polymyxins is also a current concern, especially in nephrotoxicity. Due to its pharmacokinetic advantages, PMB is preferred to be recommended in guidelines and consensus compared to CMS (Tamma et al., 2024; Tsuji et al., 2019). However, the nephrotoxicity of PMB remains a significant concern in clinical applications. Previous meta-analysis has indicated that the incidence of AKI associated with PMB is as high as 38% (Sisay et al., 2021). The high incidence of AKI hinders the widespread use of PMB, requiring the urgent need for finding safe alternatives in clinical practice. When CS or PMB was utilized to treat infections for critically ill patients (Yang et al., 2024), the incidence of AKI in the PMB group was significantly higher than that in the CS group under the condition of the unmatched cohort (20.8% vs. 9.0%, *p* = 0.002) and the matched cohort (21.1% vs. 7.0%, *p* = 0.004). This similar trend was also observed in another real-world study (Zhang et al., 2024). In our study, the CS group exhibited a significantly lower incidence of AKI before and after IPTW adjustment, which was consistent with the reported study. The reason may be attributed to structural differences, leading to varying affinities for cell membranes and distinct toxicity profiles (Zhang et al., 2024). Additionally, it is important to note that one patient exhibited skin pigmentation in the CS group. The ADR of pigmentation is mostly associated with PMB therapy in the previous reported study. The discrepancies in the incidence of skin pigmentation between CS and PMB may be due to the date of their availability on the market related to the number of studies. Therefore, we should also pay attention to the incidence of pigmentation in the future when CS therapy is adopted. Since the antibiotic resistance of CRAB challenged the clinical treatment strategies, the lower incidence of AKI associated with CS suggests that CS may be a safer option, particularly for patients with pre-existing renal impairment or a high risk of nephrotoxicity.

There are also some limitations to our study. There is some potential bias due to the design of a retrospective cohort since unmeasured confounders could not be completely despite the use of IPTW. Furthermore, the study conducted in three hospitals in a single province of China may limit generalizability to other regions or countries due to the local epidemiology of CRAB, antibiotic prescribing practices, and healthcare resources. The characteristics of CS and PMB formulations used in China may differ from those available in other countries. Meanwhile, the assessment of neurotoxicity and skin pigmentation may be subjective, and there may have been inconsistencies in how these ADRs were diagnosed and reported. There is also some potential impact of co-resistance to other antibiotics. CRAB strains may have varying resistance profiles beyond carbapenems, which could affect treatment outcomes. Prospective cohort studies should be conducted to confirm the results of the study. Additionally, dosage regimens and dose adjustments are determined by the clinician. Not all patients receive a loading dose, and the daily dose is generally conservative for safety and economic reasons. PMB and CS concentrations in the serum were not determined in this study. In future prospective cohort studies, it is necessary to establish standardized dosing regimens for CS and PMB and evaluate their efficacy and safety under standardized dosing conditions.

## Conclusion

We conducted a retrospective cohort study to evaluate the efficacy and safety of CS vs. PMB therapy for the given bacteria selected as CRAB. This is a multicenter retrospective cohort study examining the independent risk factor of bacterial species in a head-to-head comparison between CS and PMB. Our study indicates that CS therapy shows the same efficacy as PMB therapy, but it has a lower risk of AKI than PMB. Although our study suggests that CS may be associated with a lower risk of AKI compared to PMB in treating CRAB infections, this finding should be interpreted cautiously due to the limitations of the retrospective design. Further prospective studies are needed to confirm this observation and to assess the clinical impact of this potential safety advantage. These findings provide additional evidence to assist clinicians in making treatment decisions for CRAB infections. Future large-scale prospective clinical trials are necessary to further validate the efficacy and safety between CS and PMB.

## Data Availability

The raw data supporting the conclusions of this article will be made available by the authors, without undue reservation.
