# Supplementary material for: Colistin sulfate versus polymyxin B for the treatment of infections caused by carbapenem-resistant Acinetobacter baumannii: a multicenter retrospective cohort study
Source: Front Pharmacol. 2025 May 14;16:1540925. doi: 10.3389/fphar.2025.1540925 (PMC12116560; doi:10.3389/fphar.2025.1540925)
Supplement: Supplementary file 1 [file Table1.docx]

**Supplementary information**

**Table S1** STROBE statement-checklist of items that should be included in reports of observational studies.

**Table S2** Other characteristics of patients.

**Table S3** Stage of AKI severity, duration, and rates of RRT application.

**Table S4** Absolute risk reduction and number needed to harm for AKI.

**Table S1** STROBE statement-checklist of items that should be included in reports of observational studies.

|  | Item No | Recommendation | Reported on Page  Number/Line Number | Reported on  Section/Paragraph |
| --- | --- | --- | --- | --- |
| Title and abstract | 1 | (a) Indicate the study’s design with a commonly used term in the title or the abstract | 1/1 | Title |
|  |  | (b) Provide in the abstract an informative and balanced summary of what was done and what was found | 2/7-22 and 3/1 | Abstract |
| **Introduction** | | |  |  |
| Background/rationale | 2 | Explain the scientific background and rationale for the investigation being reported | 4/2-15 | Introduction/ Paragraph 1 |
| Objectives | 3 | State specific objectives, including any prespecified hypotheses | 6/8-13 | Introduction/ Paragraph 5 |
| **Methods** | | |  |  |
| Study design | 4 | Present key elements of study design early in the paper | 6/16-18 | Methods/Paragraph 1 |
| Setting | 5 | Describe the setting, locations, and relevant dates, including periods of recruitment, exposure, follow-up, and data collection | 6/16-22 | Methods/Paragraph 1 |
| Participants | 6 | (a) **Cohort study**—Give the eligibility criteria, and the sources and methods of selection of participants. Describe methods of follow-up  **Case-control study**—Give the eligibility criteria, and the sources and methods of case ascertainment and control selection. Give the rationale for the choice of cases and controls.  **Cross-sectional study**—Give the eligibility criteria, and the sources and methods of selection of participants | 6/17-22 and 7/1-8 | Methods/Paragraph 2 |
|  |  | (b) **Cohort study**—For matched studies, give matching criteria and number of exposed and unexposed  **Case-control study**—For matched studies, give matching criteria and the number of controls per case | None | None |
| Variables | 7 | Clearly define all outcomes, exposures, predictors, potential confounders, and effect modifiers. Give diagnostic criteria, if applicable. | 7/9-14，21-22; 8/1-22 and 9/1-3 | Methods/Paragraph 3, 5 to 8 |
| Data sources/  measurement | 8* | For each variable of interest, give sources of data and details of methods of assessment (measurement). Describe comparability of assessment methods if there is more than one group | 7/21-22 and 8/1-7 | Methods/Paragraph 5 |
| Bias | 9 | Describe any efforts to address potential sources of bias | 9/11-12 | Methods/Paragraph 9 |
| Study size | 10 | Explain how the study size was arrived at | 10/3-5 | Results/Paragraph 1 |
| Quantitative variables | 11 | Explain how quantitative variables were handled in the analyses. If applicable, describe which groupings were chosen and why | 9/5-6, 8-9 | Methods/Paragraph 9 |
| Statistical  methods | 12 | (a) Describe all statistical methods, including those used to control for confounding | 9/5-22 | Methods/Paragraph 9 |
|  |  | (b) Describe any methods used to examine subgroups and interactions | None | None |
|  |  | (c) Explain how missing data were addressed | None | None |
|  |  | (d) **Cohort study**—If applicable, explain how loss to follow-up was addressed  **Case-control study**—If applicable, explain how matching of cases and controls was addressed  **Cross-sectional study**—If applicable, describe analytical methods taking account of sampling strategy | None | None |
|  |  | (e) Describe any sensitivity analyses | None | None |
| **Results** | | |  |  |
| Participants | 13* | (a) Report numbers of individuals at each stage of study—eg numbers potentially eligible, examined for eligibility, confirmed eligible, included in the study, completing follow-up, and analysed | 10/3-5 | Results/Paragraph 1 |
|  |  | (b) Give reasons for non-participation at each stage | 10/3-5 | Results/Paragraph 1 |
|  |  | (c) Consider use of a flow diagram | 10/5 | Results/Paragraph 1 |
| Descriptive data | 14* | (a) Give characteristics of study participants (eg demographic, clinical, social) and information on exposures and potential confounders | 10/5-22 and 11/1-5 | Results/Paragraph 1 |
|  |  | (b) Indicate number of participants with missing data for each variable of interest | None | None |
|  |  | (c) **Cohort study**—Summarise follow-up time (eg, average and total amount) | 11/7-12 | Results/Paragraph 2 |
| Outcome data | 15* | **Cohort study**—Report numbers of outcome events or summary measures over time | 11/7-12 | Results/Paragraph 2 |
|  |  | **Case-control study**—Report numbers in each exposure category, or summary measures of exposure | None | None |
|  |  | **Cross-sectional study**—Report numbers of outcome events or summary measures | None | None |
| Main results | 16 | (a) Give unadjusted estimates and, if applicable, confounder-adjusted estimates and their precision (eg, 95% confidence interval). Make clear which confounders were adjusted for and why they were included | 10/15-21; 11/7-22 and 12/1-22 | Results/Paragraph 1 and 2 |
|  |  | (b) Report category boundaries when continuous variables were categorized | None | None |
|  |  | (c) If relevant, consider translating estimates of relative risk into absolute risk for a meaningful time period | 12/20-22 | Results/Paragraph 4 |
| Other analyses | 17 | Report other analyses done—eg analyses of subgroups and interactions, and sensitivity analyses | None | None |
| Discussion | | |  |  |
| Key results | 18 | Summarise key results with reference to study objectives | 13/14-17 | Discussion/ Paragraph 2 |
| Limitations | 19 | Discuss limitations of the study, taking into account sources of potential bias or imprecision. Discuss both direction and magnitude of any potential bias | 16/11-22 and 17/1-4 | Discussion/ Paragraph 5 |
| Interpretation | 20 | Give a cautious overall interpretation of results considering objectives, limitations, multiplicity of analyses, results from similar studies, and other relevant evidence | 17/12-16 | Conclusion |
| Generalisability | 21 | Discuss the generalisability (external validity) of the study results | 17/12-19 | Conclusion |
| Other information | | |  |  |
| Funding | 22 | Give the source of funding and the role of the funders for the present study and, if applicable, for the original study on which the present article is based | None | None |

*Give information separately for cases and controls in case-control studies and, if applicable, for exposed and unexposed groups in cohort and cross-sectional studies

**Table S2** Other characteristics of patients.

| Characteristics | CS  (*n* = 58) | PMB  (*n* = 82) | *p* Value |
| --- | --- | --- | --- |
| Hospital length of stay, median (IQR) | 17 (9-23) | 17 (10-30) | 0.295 |
| Other co-pathogens | 13 (22.4%) | 24 (29.3%) | 0.438 |
| CRKP | 3 (5.2%) | 5 (6.1%) |  |
| ESBL-KP | 2 (3.4%) | 2 (2.4%) |  |
| *Klebsiella pneumoniae^a^* | 2 (3.4%) | 2 (2.4%) |  |
| CRPA | 3 (5.2%) | 4 (4.9%) |  |
| *Pseudomonas aeruginosa^b^* | 1 (1.7%) | 4 (4.9%) |  |
| CREC | 1 (1.7%) | 2 (2.4%) |  |
| *Stenotrophomonas maltophilia* | 1 (1.7%) | 3 (3.7%) |  |
| *Burkholderia cepacia* | 0 (0) | 1 (1.2%) |  |
| MRSA | 1 (1.7%) | 1 (1.2%) |  |
| MSSA | 2 (3.4%) | 1 (1.2%) |  |
| MIC distribution |  |  |  |
| MIC ≤ 0.25 μg/ml | 32 (55.2%) | 43 (52.4%) | 0.864 |
| MIC = 0.5 μg/ml | 13 (22.4%) | 14 (17.1%) | 0.515 |
| MIC = 1 μg/ml | 11 (19.0%) | 20 (24.4%) | 0.537 |
| MIC = 2 μg/ml | 2 (3.4%) | 5 (6.1%) | 0.699 |
| Nebulized polymyxin | 2 (3.4%) | 5 (6.1%) | 0.753 |
| CRRT | 23 (39.7%) | 26 (31.7%) | 0.371 |
| ARDS | 19 (32.8%) | 15 (18.3%) | 0.071 |
| Septic Shock | 16 (27.7 %) | 24 (29.3%) | 0.852 |

^a^ Non-β-lactamase-producing *Klebsiella pneumoniae*.

^b^ Non-β-lactamase-producing *Klebsiella pneumoniae.*

Abbreviations: CS, colistin sulfate; PMB, polymyxin B; CRKP, carbapenem-resistant *Klebsiella pneumoniae;* ESBL-KP, extended-spectrum β-lactamase-producing *Klebsiella pneumoniae;* CRPA, carbapenem-resistant *Pseudomonas aeruginosa;* CREC, carbapenem-resistant *Escherichia coli;* MRSA, methicillin-resistant *Staphylococcus aureus;* MSSA, methicillin-sensitive *Staphylococcus aureus;* CRRT, continuous renal replacement therapy; ARDS, acute respiratory distress syndrome.

**Table S3** Stage of AKI severity, duration, and rates of RRT application.

| AKI | CS  (*n* = 58) | PMB  (*n* = 82) | *p* Value |
| --- | --- | --- | --- |
| Overall | 3 (5.2%) | 16 (19.5%) | 0.023 |
| Stage 1^#^ | 1 (1.7%) | 6 (7.3%) | / |
| Stage 2^#^ | 1 (1.7%) | 7 (8.5%) | / |
| Stage 3^#^ | 1 (1.7%) | 3 (4.9%) | / |
| Need for RRT | 1 (1.7%) | 3 (3.6%) | 0.530 |
| Duration of AKI, mean (SD) | 6.3 (3.5) | 6.5 (4.6) | 0.592 |

**^#^** AKI was staged for severity according to the Kidney Diseases Improving Global Outcomes criteria. Abbreviations: CS, colistin sulfate; PMB, polymyxin B; AKI, acute kidney injury; RRT, renal replacement therapy.

Table S4 Absolute risk reduction and number needed to harm for AKI.

|  | Incidence of AKI | |  | ARR |  | NNH |
| --- | --- | --- | --- | --- | --- | --- |
|  | PMB | CS |  | 95% CI |  | 95% CI |
| Before IPTW | 19.5% (16/82) | 5.2% (3/58) |  | 0.14 (0.04-0.25) |  | 7 (4-25) |
| After IPTW | 18.5% (15/80) | 5.3% (3/56) |  | 0.13 (0.03-0.24) |  | 8 (4-34) |

Abbreviations: CS, colistin sulfate; PMB, polymyxin B; AKI, acute kidney injury; ARR, absolute risk reduction; NNH, number needed to harm; IPTW, inverse probability of treatment weighting.
